# Sequential pre-disinfection with chlorhexidine and alcohol reduces periprosthetic joint infection after primary knee arthroplasty: A case-control study

**DOI:** 10.1097/MD.0000000000036101

**Published:** 2023-11-24

**Authors:** Jinhao Zhang, Chenjie Xia, Junhui Zhang, Shicheng Wang, Jin Li

**Affiliations:** a Department of Orthopedic Surgery, The Affiliated Lihuili Hospital of Ningbo University, Ningbo, China; b Department of Orthopedic Surgery, Ningbo No. 6 Hospital, Ningbo, China.

**Keywords:** alcohol, chlorhexidine, knee arthroplasty, periprosthetic joint infection

## Abstract

A retrospective case-control study was conducted to assess whether patients who underwent sequential preoperative disinfection before primary total knee or unicompartmental arthroplasty had a lower rate of postoperative infection than those who did not. In our study, 1025 patients who underwent total knee or unicompartmental arthroplasty at 2 medical centers between September 1, 2020, and August 31, 2021, were enrolled. Statistical analysis was performed for 976 cases, including 966 and 10 uninfected and infected cases, respectively. All patients were followed up for 1-year. Data analysis was performed by binary logistic regression and adjusted for 2 confounding factors: general anesthesia and rheumatoid arthritis. IBM SPSS for Windows (version 25.0; IBM Co., Armonk, NY) software was used to perform all statistical analyses. During the study period, of the 976 patients, 10 cases of infections were detected. Sequential pre-disinfection (adjusted odds ratio 0.14, 95% confidence interval: 0.03–0.54, *P* = .005) could reduce the incidence of infection. Based on the results of this study, bathing the whole lower limb with 2% chlorhexidine on the night before surgery followed by 70% alcohol application 1 hour before surgery is effective for preventing periprosthetic joint infection during primary total knee or unicompartmental arthroplasty.

## 1. Introduction

Knee osteoarthritis (KOA) is a frequent joint disease worldwide due to aging.^[1,2]^ Knee arthroplasty (KA) is commonly used to treat advanced symptomatic KOA, and over 90% of these patients show satisfactory clinical outcomes.^[3]^ Periprosthetic joint infection (PJI) is a devastating complication of KA leading to joint disability, decline in the quality of life, and early surgical revision. The occurrence of PJI is estimated between 0.8% and 1.9% in patients with primary KA, and the infection occurs majorly within 1-year post-surgery (No. 2023241). Despite the relatively low occurrence rate, the cure of PJI is extremely difficult and costly. Patients with PJI are on a long antimicrobial treatment regimen and suffer multiple debridements, bringing a huge financial burden on the family with excessive demand for social, medical, and healthcare resources. Therefore, preventing PJI after KA is important.

PJI is caused by the introduction of the colonized bacterium into the surgical site through the bloodstream or adjacent tissue spread.^[4,5]^ Several factors contribute to the occurrence of PJI, and most of them are unmodifiable including advanced age, obesity, chronic liver disease, nutrition condition, and surgical time.^[6]^ As a significant modifiable intervention, preoperative skin preparation has prominent advantages in preventing PJI, as it can directly inactivate colonized bacteria in the surgical site. Both chlorhexidine and ethyl alcohol are potent biocides against a broad-spectrum of bacteria. Massive clinical evidence revealed that bathing or showering with chlorhexidine shows a higher reduction in skin bacterial load compared to soap.^[7]^ Pre-disinfection with chlorhexidine can reduce the incidence of PJI.^[8]^ Moreover, 70% alcohol is mixed into a chlorhexidine/iodine solution to improve antibacterial efficiency. Nevertheless, a few studies reported opposite results, whereby pre-disinfection with chlorhexidine or alcohol showed no significant effects on the prevention of PJI. Thus, high-quality clinical research to standardize pre-disinfection protocols is necessitated.

The purpose of this case-control study was to assess the effects of bathing with 2% chlorhexidine the night before surgery and 70% alcohol application, 1 hour before surgery (i.e., sequential pre-disinfection with chlorhexidine and alcohol) to prevent PJI after primary total knee or unicompartmental arthroplasty.

## 2. Materials and Methods

### 2.1. Study participants

**Figure 1.**
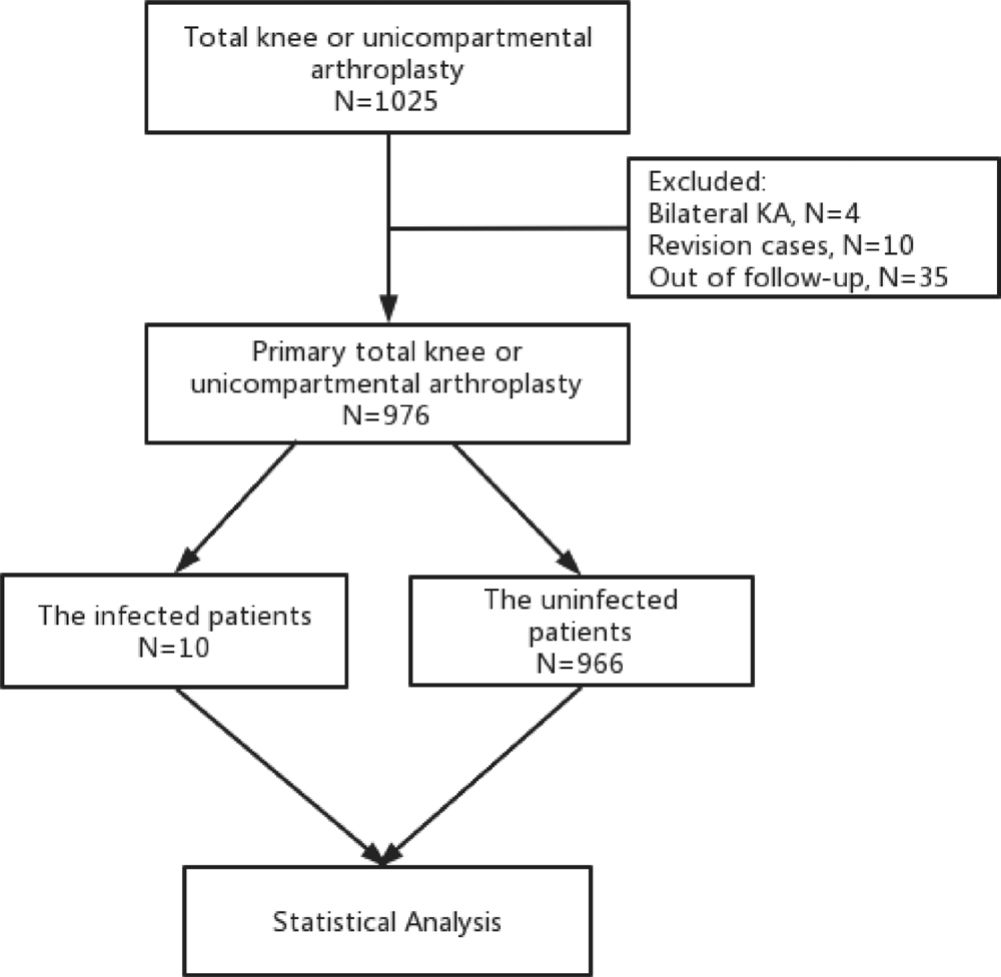
The flowchart of primary total knee or unicompartmental arthroplasty case enrollment.

This retrospective case-control study was approved by the Ethics Committee and Research Commission of Lihuili Hospital affiliated with Ningbo University (No. 2023241). A total of 1025 patients who underwent total knee or unicompartmental arthroplasty at 2 medical centers (Lihuili Hospital affiliated with Ningbo University and Ningbo No. 6 Hospital) from September 1, 2020, to August 31, 2021, were enrolled. The inclusion criteria of this study included KOA patients with independent living ability, age from 18 to 80 years, over 1 year of follow-up post-surgery, and availability of informed consent. Exclusion criteria were previous native knee infection, KA for trauma-related reasons, bilateral surgery, revision cases, and allergy to chlorhexidine or alcohol. According to these screening criteria, 976 patients were finally identified with 35 cases excluded due to lack of follow-up; 10 were revision cases, and 4 were cases of bilateral KA, as shown in Figure 1. Information of patient characteristics including age, body mass index (BMI) and sex, sequential pre-disinfection with or without chlorhexidine and alcohol, anesthesia methods, smoking, preoperative blood glucose, preoperative albumin, hypertension, coronary heart disease, diabetes, hyperlipidemia, chronic hepatitis B, and rheumatoid arthritis were obtained from the medical records.

### 2.2. Pre-disinfection protocol and surgical management

Sequential pre-disinfection involved bathing the whole lower limb with 2% chlorhexidine on the night before surgery followed by 70% alcohol application, 1 hour before surgery, while washing with soap was performed in other cases. General or lumbar anesthesia was administered according to each patient’s conditions and choice. After the tourniquet was applied to the thigh with pressure maintained at 280 mm Hg, routine disinfection with 5% iodine was performed on the lower limb underneath the tourniquet. All surgeries were performed through a medial parapatellar approach that incised the quadriceps tendon along its medial border. After tibial and femoral osteotomy induced by guide plates, the prosthesis (NexGen, ZIMMER) was implanted and fixed by bone cement. Patients typically did not receive catheterization before surgery and wound drainage after surgery. Painkillers, antibiotics, anticoagulants, and wound dressing with 5% iodine were used routinely to alleviate pain and prevent postoperative infection and thrombosis.

### 2.3. Definition of PJI

The diagnosis of PJI is based on international consensus as follows: (1) a sinus tract communicating with the prosthesis or (2) isolation of pathogens from at least 2 separate tissue or liquid samples obtained from the affected prosthetic joint by culture or (3) 1 of the following 6 criteria can be established: elevated erythrocyte sedimentation rate and C-reactive protein, elevated synovial leukocyte, elevated synovial polymorphonuclear leukocytes percentage, presence of purulence in the affected joint, isolate microorganisms in a periprosthetic tissue or fluid culture, and/or more than 5 neutrophils in 5 high-power fields observed on histological analysis of the tissue surrounding the prosthesis at 400-fold.^[9]^ PJI is defined as an infection occurring up to 1-year after surgery in patients receiving implants and affecting deep tissue at the operation site. Therefore, patients were followed up for 1-year after surgery and superficial surgical site infections were excluded from analysis.

### 2.4. Statistical analysis

All data were analyzed using IBM SPSS for Windows (version 25.0; IBM Co., Armonk, NY). Continuous data, such as patient age, BMI, blood glucose, and albumin, were expressed as the mean and standard deviation; categorical data, such as patient sex, sequential pre-disinfection with or without chlorhexidine and alcohol, anesthesia methods, smoking, hypertension, coronary heart disease, diabetes, hyperlipidemia, chronic hepatitis B, and rheumatoid arthritis, were expressed in numbers and percentages. Differences in continuous data between infected and uninfected patients were assessed using the Student *t* test or the Mann–Whitney test, and the categorical data of the 2 groups were compared using Fisher exact test and the Chi-squared test. Finally, binary logistic regression analysis was used to control confounding factors. Odds ratios (ORs) and 95% confidence intervals (CIs) were computed to assess the strength of associations between sequential pre-disinfection and PJI. Statistical significance was defined as *P* < .05.

## 3. Results

To identify the risk factors of PJI, 10 patients with PJI and 966 uninfected patients who underwent primary knee or unicompartmental arthroplasty at 2 medical centers in Ningbo were included in a case-control study. Their baseline information (age, sex, and BMI) and potential influencing factors including with or without sequential pre-disinfection, anesthesia method, smoking, blood glucose, albumin, hypertension, coronary heart disease, diabetes, hyperlipidemia, chronic hepatitis B, and rheumatoid arthritis were collected (Table 1). Differences in age, sex, and BMI between the 2 groups were not statistically significant. Compared to uninfected patients, patients with PJI after KA had higher presence rates in general anesthesia (40% vs 11.80%) and rheumatoid arthritis (10% vs 0.83%), consistent with previous reports.^[10,11]^ Moreover, a lower frequency of pre-disinfection in patients with PJI (40% vs 86.85%) indicates that the sequential pre-disinfection method could significantly reduce the risk of PJI after KA. As sequential pre-disinfection is a simple clinical intervention that is rarely reported and may lead to positive clinical outcomes, the relationship between sequential pre-disinfection and PJI needs to be assessed.

**Table 1:** Comparison of demographic factors between infected/noninfected group.

	Uninfected (n = 966)	Infected (n = 10)	*P* value
Age, yr	68.04 ± 7.27	68.40 ± 7.50	.877
BMI, kg/m²	25.78 ± 3.56	25.73 ± 2.74	.967
Female sex	694 (71.84%)	7 (70.00%)	.897
Preoperative disinfection	839 (86.85%)	4 (40.00%)	<.001
General anesthesia	114 (11.80%)	4 (40.00%)	.007
Smoker	49 (5.07%)	1 (10.00%)	.482
Blood glucose, mmol/L	6.81 ± 2.32	7.14 ± 2.27	.543
Albumin, g/L	43.73 ± 3.68	42.64 ± 4.91	.546
Hypertension	531 (54.97%)	6 (60.00%)	.750
Coronary heart disease	18 (1.86%)	0 (0.00%)	.663
Diabetes	149 (15.42%)	1 (10.00%)	.636
Hyperlipidemia	84 (8.70%)	0 (0.00%)	.329
Chronic hepatitis B	6 (0.62%)	0 (0.00%)	.759
Rheumatoid arthritis	8 (0.83%)	1 (10.00%)	.003

Continuous variables are presented as mean (standard deviation) and categorical variables are presented as raw number (%).

BMI = body mass index.

Risk factors for developing PJI in the univariate analysis included the absence of sequential pre-disinfection (*P* < .001), general anesthesia (*P* = .007), and rheumatoid arthritis (*P* = .003) (Table 2). Primary analysis revealed that patients who underwent sequential pre-disinfection had a 90% lower risk of infection than patients who did not (OR = 0.10, 95% CI: 0.03–0.36). To study the effect of sequential pre-disinfection on the incidence of PJI, we performed binary logistic regression and adjusted for possible confounding factors (Table 3). After controlling for 2 influencing factors of general anesthesia and rheumatoid arthritis, pre-disinfection remained an independent protective factor for postoperative infection (adjusted OR 0.14, 95% CI: 0.03–0.54). Therefore, our results demonstrate that the use of 2% chlorhexidine on the whole lower limb on the night before surgery and the application of 70% alcohol an hour before the operation significantly affect PJI.

**Table 2:** Variables associated with the infection by univariate analysis.

	OR	95% CI	*P* value
Age	1.01	(0.92, 1.10)	.877
BMI	1.00	(0.84, 1.19)	.967
Female	0.91	(0.23, 3.56)	.898
Preoperative disinfection	0.10	(0.03, 0.36)	<.001
General anesthesia	4.98	(1.39, 17.92)	.014
Smoker	2.08	(0.26, 16.74)	.492
Blood glucose	1.05	(0.84, 1.33)	.650
Albumin	0.93	(0.79, 1.09)	.353
Hypertension	1.23	(0.34, 4.38)	.751
Coronary heart disease	0.00	(0.00, Inf)	.993
Diabetes	0.61	(0.08, 4.84)	.640
Hyperlipidemia	0.00	(0.00, Inf)	.990
Chronic hepatitis B	0.00	(0.00, Inf)	.994
Rheumatoid arthritis	13.31	(1.50, 117.71)	.020

BMI = body mass index, CI = confidence interval, OR = odds ratio.

**Table 3:** Relationship between preoperative disinfection and infection.

	Group	OR	95% CI	*P* value
Preoperative disinfection	Not adjusted	0.10	(0.03, 0.36)	<.001
	Adjusted 1	0.11	(0.03, 0.39)	<.001
	Adjusted 2	0.13	(0.03, 0.52)	.004
	Adjusted 3	0.14	(0.03, 0.54)	.005

Adjusted 1 = adjusted for rheumatoid arthritis, Adjusted 2 = adjusted for general anesthesia, Adjusted 3 = adjusted for rheumatoid arthritis and general anesthesia, CI = confidence interval, OR = odds ratio.

## 4. Discussion

The most important finding of this case-control study was that sequential pre-disinfection reduces the rate of infection after primary KA. This result is in line with some studies that suggest a lack of conclusive evidence for the reduction in the risk of PJI with chlorhexidine bathing and povidone-iodine application. Sequential pre-disinfection which plays an important role in preventing PJI is convenient to operate.

Povidone-iodine provides broad-spectrum bactericidal activity with minimal toxicity.^[12]^ Therefore, several surgical disciplines have advocated wound irrigation with diluted povidone-iodine to reduce postoperative infection rates.^[13]^ Due to its wide application in surgery, this antiseptic has been incorporated into recent clinical practice guidelines by the Centers for Disease Control and Prevention.^[14]^ The superior efficacy of chlorhexidine compared with povidone-iodine in reducing bacterial flora has been demonstrated in several studies on presurgical preparation.^[15]^ The cumulative effect of extra washings with chlorhexidine has been noted when this antiseptic is used to wash hands. Consequently, the simultaneous use of 2 antiseptics can increase the disinfectant effect. In our study, we found that sequential disinfection with chlorhexidine and povidone-iodine before operation was associated with a significantly lower rate of PJI compared to controls.

Preoperative disinfection has been proposed as a preventive measure to reduce PJI in KA. Eiselt compared the incidence of surgical site infection in 727 patients who were instructed to use a povidone-iodine wash before the process with 736 patients with total joint arthroplasty who were instructed to use a chlorhexidine cloth to disinfect the surgical site the night before and the morning of the operation. Perioperative chlorhexidine rinses in patients undergoing surgery decreased the infectious complications to 1.59% versus 3.19% in the control group.^[16]^ Michael reported similar findings as Eiselt, the infection rate was 3% in 711 cases, while there was no surgical site infection in 136 cases following the advanced preoperative preparation protocol, and only 1 patient (1.5%) among 65 showed infection who partially followed the advanced protocol.^[8]^ Allen B. Kaiser^[7]^ reported that the use of chlorhexidine resulted in a reduction in baseline counts in the subclavian and inguinal sites relative to staphylococcal flora. In this study, the colony numbers of chlorhexidine and povidone-iodine groups decreased, further demonstrating our view.

The main limitation of our study is information bias, given its retrospective case-control design with data collected from medical records, the demographic data including tourniquet time and surgery time were not available for all patients. The study had a limited sample size as it was focused solely on primary patients with KA, which may have caused the outcomes of this study to be ineffective. It is a dual-center study, making it difficult to consider technical variations among all surgeons over its course and its impact on the rate of PJI. Consequently, future prospective cohort studies with a larger sample size and a single-center study may be needed to confirm the findings of this study. Finally, limitations to our study also include the culture of microorganisms for comparison preoperatively, during, and postoperatively in surgical areas, which may demonstrate our view.

Despite these limitations, our findings suggest that sequential pre-disinfection before surgery has a lower postoperative infection rate. This simple protocol addresses the issue of patient compliance and can reduce morbidity, mortality, and the financial burden of managing periprosthetic infection, which is simple to operate. We hope that the benefits of this method of sequential pre-disinfection can be expanded to other types of surgery and warrant further research.

## 5. Conclusion

PJI remains the most devastating and costly complication after KA. Sequential pre-disinfection with a bath of 2% chlorhexidine on the whole lower limb on the night before surgery followed by 70% alcohol application 1 hour before surgery reduces the incidence of infection after primary KA.

## Author contributions

**Conceptualization:** Shicheng Wang.

**Data curation:** Jinhao Zhang, Shicheng Wang.

**Formal analysis:** Chenjie Xia.

**Investigation:** Chenjie Xia.

**Methodology:** Junhui Zhang, Shicheng Wang.

**Project administration:** Junhui Zhang, Jin Li.

**Resources:** Junhui Zhang, Jin Li.

**Software:** Jinhao Zhang, Shicheng Wang, Jin Li.

**Supervision:** Chenjie Xia, Jin Li.

**Validation:** Chenjie Xia.

**Visualization:** Jinhao Zhang, Chenjie Xia, Jin Li.

**Writing – original draft:** Jinhao Zhang.

**Writing – review & editing:** Shicheng Wang.
